# Astaxanthin overproduction in yeast by strain engineering and new gene target uncovering

**DOI:** 10.1186/s13068-018-1227-4

**Published:** 2018-08-23

**Authors:** Jin Jin, Ying Wang, Mingdong Yao, Xiaoli Gu, Bo Li, Hong Liu, Mingzhu Ding, Wenhai Xiao, Yingjin Yuan

**Affiliations:** 1 Key Laboratory of Systems Bioengineering (Ministry of Education), School of Chemical & Engineering, Tianjin University, No. 92, Weijin Road, Nankai District, Tianjin, 300072 People’s Republic of China; 2 SynBio Research Platform, Collaborative Innovation Center of Chemical Science and Engineering (Tianjin), Tianjin University, Tianjin, 300072 People’s Republic of China

**Keywords:** Metabolic engineering, Astaxanthin, *Saccharomyces cerevisiae*, ARTP mutagenesis, Novel gene targets

## Abstract

**Background:**

Astaxanthin is a natural carotenoid pigment with tremendous antioxidant activity and great commercial value. Microbial production of astaxanthin via metabolic engineering has become a promising alternative. Although great efforts have been conducted by tuning the heterologous modules and precursor pools, the astaxanthin yields in these non-carotenogenic microorganisms were still unsatisfactory for commercialization, indicating that in addition to targeted tailoring limited targets guided by rationally metabolic design, combining more globe disturbances in astaxanthin biosynthesis system and uncovering new molecular mechanisms seem to be much more crucial for further development. Since combined metabolic engineering with mutagenesis by screening is a powerful tool to achieve more global variations and even uncover more molecular targets, this study would apply a comprehensive approach integrating heterologous module engineering and mutagenesis by atmospheric and room temperature plasma (ARTP) to promote astaxanthin production in *Saccharomyces cerevisiae*.

**Results:**

Here, compared to the strain with β-carotene hydroxylase (CrtZ) from *Alcaligenes* sp. strain PC-1, involving new CrtZ from *Agrobacterium aurantiacum* enhanced astaxanthin yield to 1.78-fold and increased astaxanthin ratio to 88.7% (from 66.6%). Astaxanthin yield was further increased by 0.83-fold (to 10.1 mg/g DCW) via ARTP mutagenesis, which is the highest reported yield at shake-flask level in yeast so far. Three underlying molecular targets (*CSS1*, *YBR012W*-*B* and *DAN4*) associated with astaxanthin biosynthesis were first uncovered by comparative genomics analysis. To be noted, individual deletion of *CSS1* can recover 75.6% improvement on astaxanthin yield achieved by ARTP mutagenesis, indicating *CSS1* was a very promising molecular target for further development. Eventually, 217.9 mg/L astaxanthin (astaxanthin ratio was 89.4% and astaxanthin yield was up to 13.8 mg/g DCW) was obtained in 5-L fermenter without any addition of inducers.

**Conclusions:**

Through integrating rational engineering of pathway modules and random mutagenesis of hosts efficiently, our report stepwise promoted astaxanthin yield to achieve the highest reported one in yeast so far. This work not only breaks the upper ceiling of astaxanthin production in yeast, but also fulfills the underlying molecular targets pools with regard to isoprenoid microbial overproductions.

**Electronic supplementary material:**

The online version of this article (10.1186/s13068-018-1227-4) contains supplementary material, which is available to authorized users.

## Background

Astaxanthin, a member of carotenoid pigments with much higher antioxidant activity than other carotenoids and vitamin E, has tremendous commercial value in the aquaculture, food, cosmetic and pharmaceutical industries [1, 2]. In addition to currently commercial astaxanthin source by chemical synthesis or extraction from natural producers (such as the green algae or the red yeast) [3], microbial production of astaxanthin via metabolic engineering has emerged as a promising alternative [4, 5]. In recent years, heterologous productions of astaxanthin have been successfully achieved in *Escherichia coli* [6–10], *Saccharomyces cerevisiae* [11, 12] and *Corynebacterium glutamicum* [13] through introduction of astaxanthin biosynthesis pathway into these microorganisms. Although great efforts have been conducted by metabolically engineering the heterologous modules and precursor pools, the highest astaxanthin yield in *E. coli* or *S. cerevisiae* was 15.1 mg/g DCW [14] and 8.10 mg/g DCW [12] so far, respectively (Table 1). The astaxanthin yields in these non-carotenogenic microorganisms were still unsatisfactory for commercialization, indicating that many more biological functions, such as biosynthesis, transport, storage and even tolerance, would likely to be regulated at the same time for further higher astaxanthin yields. In other words, in addition to targeted tailoring limited points guided by rationally metabolic design, combining global disturbances in astaxanthin biosynthesis system and further uncovering new molecular mechanisms seem to be much more crucial for further development.

**Table 1:** Astaxanthin production by different microorganisms

Hosts	Fermentation level	Astaxanthin yield (mg/g DCW)	Astaxanthin ratio (%)	References
Non-carotenogenic microorganisms				
*E. coli*	Shake-flask	1.4	95	[7]
	Shake-flask	1.99	90	[6]
	Shake-flask	5.8	N.D.	[8]
	Shake-flask	7.4	96.6	[9]
	Shake-flask	8.64	N.D.	[10]
	Shake-flask	15.1	N.D.	[14]
*S. cerevisiae*	Shake-flask	4.7	N.D.	[11]
	Shake-flask	8.1	N.D.	[12]
	Shake-flask 5-L bioreactor	10.1 13.8	89.4	This study
*Kluyveromyces marxianus*	5-L bioreactor	9.97	N.D.	[5]
Carotenogenic microorganisms				
*Haematococcus pluvialis*	Shake-flask	77.2	N.D.	[3]

*N.D.* not determined

Apart from metabolic engineering strategy based on rational design, mutagenesis followed by screening is a common strategy to improve phenotypic profiles especially for high yields and growth robustness through randomly and more globally affecting microenvironment in hosts. New molecular targets identified in mutagenesis are just valuable feedback to further rational design. Among physical and chemical methods for mutagenesis [15], an atmospheric and room temperature plasma (ARTP) method has been applied for mutagenesis of various species to obtain targeted biological features [16, 17]. The ARTP mutation system can induce diverse breakage in plasmid DNA and oligonucleotides with variation of plasma dosage [18]. A mutant *Blakeslea trispora* was isolated from samples treated with ARTP, in which the lycopene accumulation was enhanced by 55% than that in the parent strain [19]. Zhao et al. [20] also reported that improved production profiles for lipids and carotenoids were obtained in oleaginous yeast *Rhodosporidium toruloides* by ARTP. Such perfect performances of ARTP in microorganism breeding and products enhancing enable it very likely to be beneficial for better astaxanthin accumulation.

**Fig. 1:**
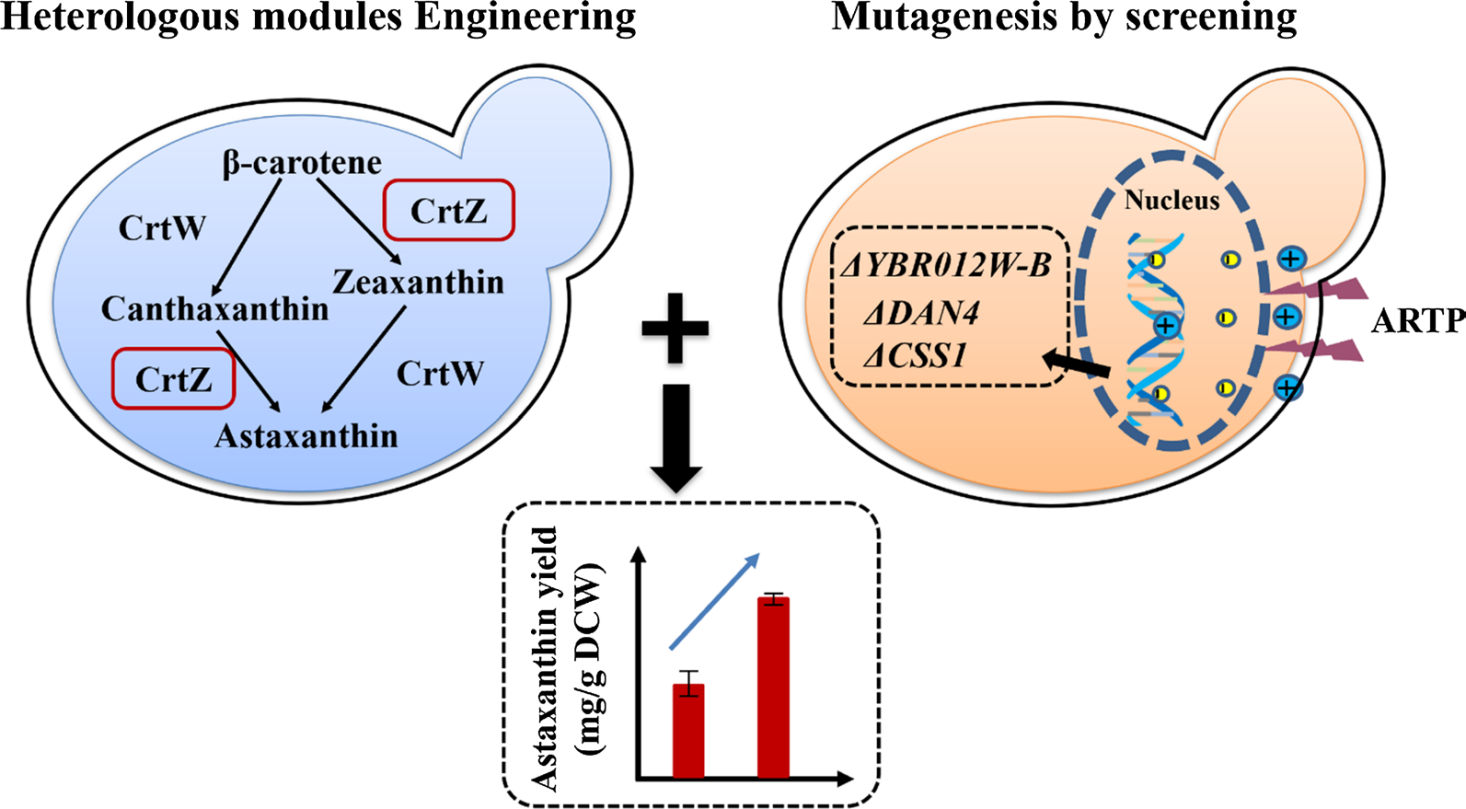
Overview of astaxanthin biosynthesis pathway from β-carotene and the strategy (heterologous modules engineering combined with mutagenesis by screening) applied in this study. The targeted protein in module engineering was boxed by red. And the gene targets, that were identified to be associated with astaxanthin biosynthesis in this research, were also presented in black round square in dashed line. ARTP, atmospheric and room temperature plasma

In the meantime, it has been reported that the limited step of astaxanthin production is the pathway from β-carotene to astaxanthin (Fig. 1), in which two enzymes, β-carotene ketolase CrtW and β-carotene hydroxylase CrtZ are required [6]. It has been revealed that many bacterial CrtWs and CrtZs could utilize β-carotene as well as its hydroxylated or ketolated products as the substrate, leading to diverse carotenoid intermediate profiles which can greatly affect astaxanthin yield and ratio [21, 22]. In our study, novel combination of CrtZ–CrtW with mutagenesis by ARTP process was employed to further enhance astaxanthin production and ratio in *S. cerevisiae* (Fig. 1). Consequently, an astaxanthin yield of 10.1 mg/g DCW (titer of 55.7 mg/L) at shake-flask level was obtained, which is the highest reported yield at shake-flask level in *S. cerevisiae*. Three promising gene targets (*CSS1*, *YBR012W*-*B* and *DAN4*) were firstly identified from ARTP mutagenesis and their influences on astaxanthin synthesis were also validated accordingly. Finally, 217.9 mg/L astaxanthin (astaxanthin ratio was 89.4% and astaxanthin yield was up to 13.8 mg/g DCW) was achieved in 5-L fermenter. Our study not only provides new gene targets for astaxanthin biosynthesis, but also highlights the importance of the combined effects of metabolic engineering and mutagenesis on microbial overproduction of natural products.

## Results and discussion

### Enhancing astaxanthin yield and ratio by involving novel combination of CrtZ–CrtW

**Fig. 2:**
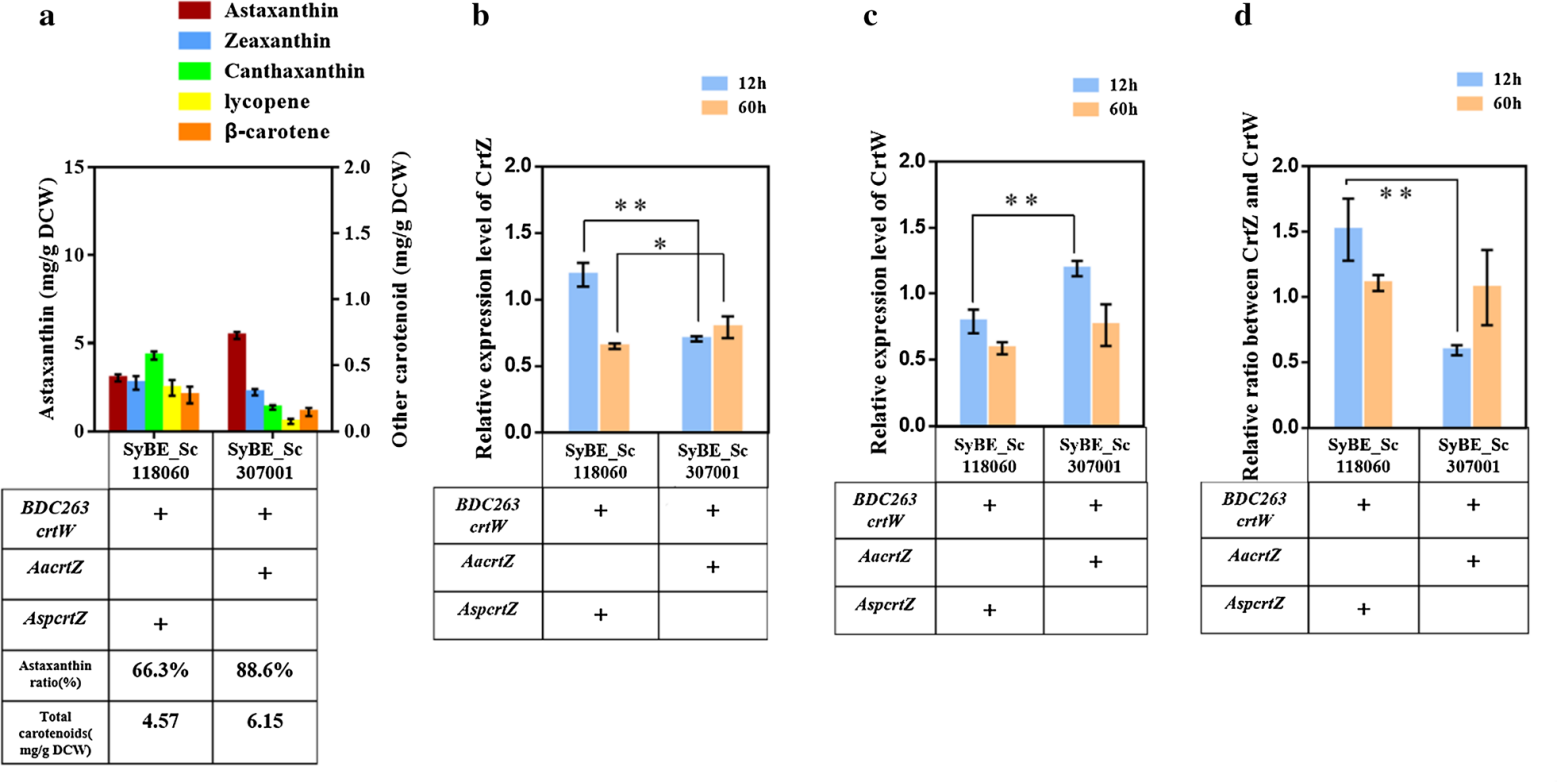
The effect of new combination of CrtZ–CrtW on astaxanthin yield and ratio. **a** Carotenoids profiles in strain SyBE_Sc118060 (with BDC263_CrtW–Asp_CrtZ) and SyBE_Sc307001 (with BDC263_CrtW–Aa_CrtZ). Strains were cultured in YPD medium for 84 h. The yield of astaxanthin and other carotenoid intermediates was analyzed by HPLC. “Astaxanthin yield” was determined as “the astaxanthin content in single cell” with unit as mg/g DCW; “astaxanthin ratio” was determined as “the astaxanthin content (mg/g DCW) to the total carotenoids content (mg/g DCW) in single cell” as %. Other carotenoids indicated zeaxanthin, canthaxanthin, lycopene and β-carotene. **b** The relative transcription level of CrtZ. **c** The relative transcription level of CrtW. **d** The relative ratio of CrtZ to CrtW. Cells were harvested at 12 h (early phase) and 60 h (late phase). The relative transcription level for each gene was determined as − ΔCt, using gene ALG9 for normalization. The relative ratio of CrtZ to CrtW was calculated as 2^−ΔCt(CrtZ)^/2^−ΔCt(CrtW)^. All the error bars represent standard deviations calculated from three independent experiments. Significant levels of *t*-test: *P < 0.05, **P < 0.01

CrtZ and CrtW are widely found in bacteria, plants, archaebacteria and other organisms [23, 24]. The combination of these two enzymes is critical to astaxanthin production [6]. Our previous study further demonstrated that CrtW is more crucial to astaxanthin accumulation than CrtZ [25]. CrtW from *Brevundimonas vesicularis* DC263 (BDC263_CrtW) has been proved as a promising ketolase exhibiting general substrate diversity [25]. Although the combination of BDC263_CrtW and Asp_CrtZ (CrtZ from *Alcaligenes* sp. strain PC-1) achieved better astaxanthin yield among our former thirty tested groups [25], canthaxanthin was still accumulated as the major intermediate in the fermentation product (Fig. 2a), thus improving the hydroxylation of canthaxanthin by CrtZ would be beneficial for further promoting astaxanthin yield and ratio here.

Fraser et al. [26] once compared the catalytic activity of *E. coli*-purified Aa_CrtZ (CrtZ from *Agrobacterium aurantiacum*) and Asp_CrtZ. As a result, Aa_CrtZ showed 2.6-fold enzyme activity of Asp_CrtZ with β-carotene as the substrate (9.0–3.4 pmol/h/mg protein) and 2.1-fold enzyme activity of Asp_CrtZ using canthaxanthin as the substrate (60–28 pmol/h/mg protein). Consistently, Aa_CrtZ achieved 68.8% increase on zeaxanthin accumulation than Asp_CrtZ, when these two enzymes were individually expressed in our β-carotene producer SyBE_Sc118030 (Additional file 1: Figure S2). Therefore, in this study, the novel combination of CrtZ–CrtW (Aa_CrtZ–BDC263_CrtW) was adopted and expressed (Additional file 1: Figure S1) in our existing high β-carotene producer SyBE_Sc118030, gaining strain SyBE_Sc307001 (Table 2). Meanwhile, Real-Time PCR was conducted to investigate the transcriptional level of CrtZ and CrtW in strains SyBE_Sc118060 and SyBE_Sc307001, and SyBE_Sc118060. Samples were harvested at 12 h (early phase) and 60 h (late phase) during the cultivation. As shown in Fig. 2a and Additional file 1: Figure S2, the new combination achieved much less intermediate accumulation than the former one. To be noted, the accumulation of lycopene (peak IV, Additional file 1: Figure S2), β-carotene (peak V, Additional file 1: Figure S2) and canthaxanthin (peak III, Additional file 1: Figure S2) was reduced by 0.773-, 0.423- and 0.694-fold, respectively (Fig. 2a). The accumulation of canthaxanthin depends on the reaction competitiveness, including the efficiency of CrtW to generate canthaxanthin from β-carotene as well as the efficiency of CrtZ to convert canthaxanthin to astaxanthin. Less canthaxanthin accumulation in strain SyBE_Sc307001 during time course might be caused by the increase in the relative transcriptional level of Aa_CrtZ to CrtW as times went on (Fig. 2b–d). Accordingly, the yield of astaxanthin (peak I, Additional file 1: Figure S2) was increased from 3.1 mg/g DCW to 5.5 mg/g DCW in strain SyBE_Sc307001 compared with strain SyBE_Sc118060 (Fig. 2a). In the meanwhile, the astaxanthin ratio was also enhanced from 66.6 to 88.7% (Fig. 2a). Therefore, strain SyBE_Sc307001 was chosen as the starting strain to generate ARTP mutagenesis library.

**Table 2:** *S. cerevisiae* strains used in this study

Strains	Description	Sources
BY4742	*MATα*, *HIS3Δ1*, *LEU2Δ0*, *LYS2Δ0*, *URA3Δ0*	[48]
SyBE_Sc118030	β-carotene-producing strain	[26]
SyBE_Sc118060	Expression of BDC263_CrtW and Asp_CrtZ (ADH1t-*Aa_crtZ*-FBA1p-TDH3p-*BDC263_crtW*-TDH2t) in strain SyBE_Sc118030	[26]
SyBE_Sc307001	Introducing plasmid BDC263_CrtW and Aa_CrtZ (ADH1t-*Aa_crtZ*-FBA1p-TDH3p-*BDC263_crtW*-TDH2t) into strain SyBE_Sc118030	This study
SyBE_Sc307121	Introducing plasmid pRS425K-Asp_CrtZ (FBA1p -*Asp*_*crtZ*-ADH1t) in strain SyBE_Sc118030	This study
SyBE_Sc307122	Introducing plasmid pRS425K-Aa_CrtZ (FBA1p-*Aa_crtZ*-ADH1t) into strain SyBE_Sc118030	This study
SyBE_Sc2110M1	ARTP mutant strain 1 of SyBE_Sc307001	This study
SyBE_Sc2110M2	ARTP mutant strain 2 of SyBE_Sc307001	This study
SyBE_Sc2110M3	ARTP mutant strain 3 of SyBE_Sc307001	This study
SyBE_Sc2110M4	ARTP mutant strain 4 of SyBE_Sc307001	This study
SyBE_Sc2110M5	ARTP mutant strain 5 of SyBE_Sc307001	This study
SyBE_Sc2110M6	ARTP mutant strain 6 of SyBE_Sc307001	This study
SyBE_Sc2110M7	ARTP mutant strain 7 of SyBE_Sc307001	This study
SyBE_Sc307104	SyBE_Sc307001, *∆FLO9::KanMX*	This study
SyBE_Sc307105	SyBE_Sc307001, *∆YLR410W*-*B::KanMX*	This study
SyBE_Sc307106	SyBE_Sc307001, *∆YBR012W*-*B::KanMX*	This study
SyBE_Sc307108	SyBE_Sc307001, *∆DAN4::KanMX*	This study
SyBE_Sc307109	SyBE_Sc307001, *∆CSS1::KanMX*	This study
SyBE_Sc307120	Integrating a *Leu2* Marker into the *HO* site of strain BY4742	[42]

### Higher astaxanthin yield and total carotenoid production achieved by ARTP mutagenesis

**Fig. 3:**
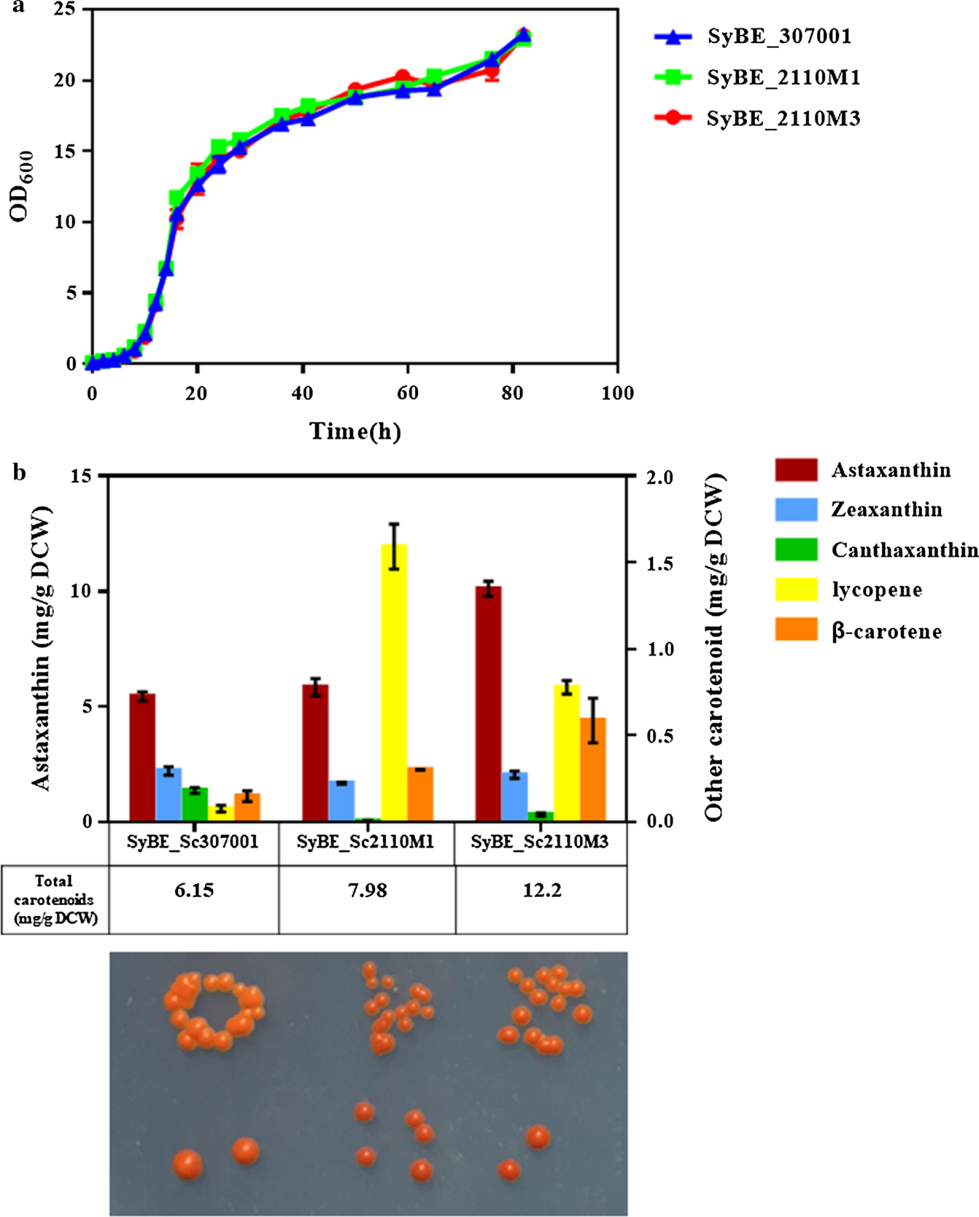
Cell growth and astaxanthin yield of ARTP mutants. Growth curve **a** and carotenoid profile **b** of strain SyBE_Sc307001 and its ARTP mutagenesis strains (SyBE_Sc2110M1 and SyBE_Sc2110M3) which achieved higher total carotenoids accumulation in YPD medium than the parent strain. A photograph was attached bellow the bar chart to illustrate visual color of the related strains. The error bars represent standard deviations calculated from duplicate experiments. “Astaxanthin yield” was determined as “the astaxanthin content in single cell” with unit as mg/g DCW. Other carotenoids indicated zeaxanthin, canthaxanthin, lycopene and β-carotene

To obtain cells with higher astaxanthin yield, strain SyBE_Sc307001 was submitted to ARTP for 30 s or 40 s individually and then grown on SD medium [27]. The death rate reached 82.6% and 86.8% under 30-s and 40-s treatments, respectively (Additional file 1: Figure S3). After radiation, seven strains exhibiting darker red pigments (SyBE_Sc2110M1-M7, Fig. 3 and Additional file 1: Figure S4) were visually picked up and then cultured to analyze their carotenoids compositions. As illustrated in Fig. 3a and Additional file 1: Figure S4a, all these mutants presented comparable cell growth to that of the control strain SyBE_Sc307001 in YPD medium [27]. In particular, astaxanthin yield in strain SyBE_Sc2110M3 was increased from 5.5 mg/g DCW to 10.1 mg/g DCW compared with the starting strain SyBE_Sc307001 (Fig. 3b), which was the highest astaxanthin yield at shake-flask level in *S. cerevisiae* to date (Table 1). Besides, more than 0.25-fold improvements on total carotenoid accumulation were also observed in strains SyBE_Sc2110M1 and SyBE_Sc2110M3 (Fig. 3b). Therefore, genomic comparison of strains SyBE_Sc2110M1, SyBE_Sc2110M3 and SyBE_Sc307001 would uncover potential gene targets contributing to higher astaxanthin yield and total carotenoid accumulation.

### Uncovering and validating gene targets contributing to higher astaxanthin yield by genomic and transcriptomics comparison

**Fig. 4:**
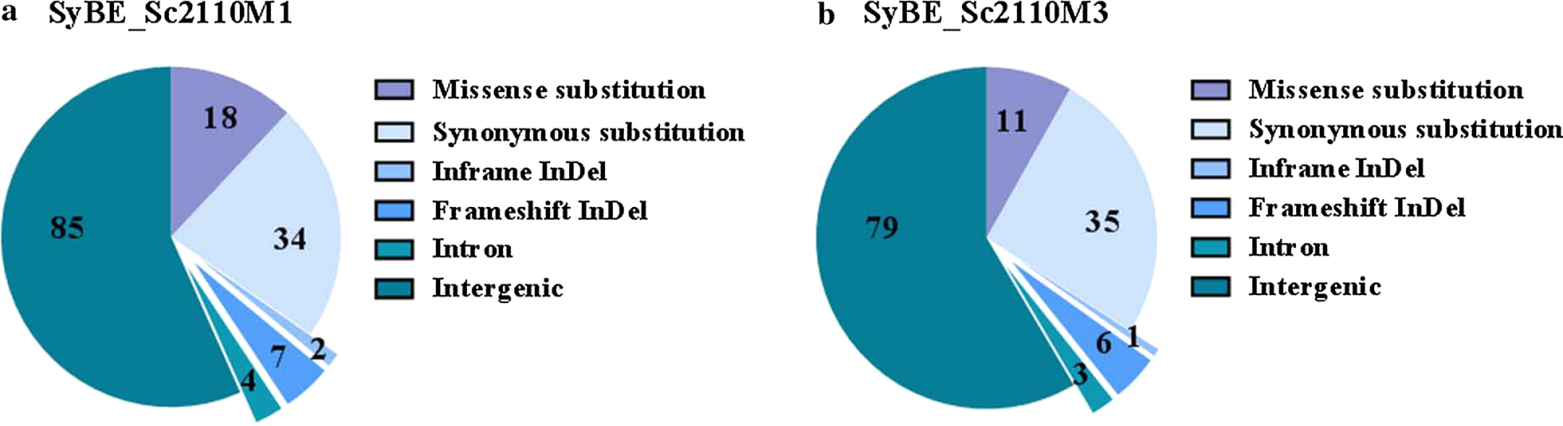
The variations in selected ARTP mutants with respect to strain SyBE_Sc307001. Variations distribution of all the variations in strain SyBE_Sc2110M1 (**a**) and SyBE_Sc2110M3 (**b**) with respect to strain SyBE_Sc307001

As showed in Additional file 1: Figure S5, the copy numbers of the plasmid carrying BDC263_CrtW–Aa_CrtZ were unchanged among strains SyBE_Sc307001, SyBE_Sc2110M1 and SyBE_Sc2110M3 which, therefore, did not contribute to the increase on astaxanthin yield by ARTP treatment. To identify the gene(s) with regard to such astaxanthin yield improvement, the whole genome of SyBE_Sc2110M1, SyBE_Sc2110M3 and SyBE_Sc307001 was re-sequenced and compared using SyBE_Sc307001 as the reference strain. As a result, there was no variation detected within gene *crtZ* and *crtW*. To date, 61 variations affecting 22 CDS and 89 variations affecting 45 intron/intergenic regions of strain SyBE_Sc2110M1 have been detected (Fig. 4a). Among these variations, there were total 27 sense mutations in CDS, containing 18 missenses, seven frame-shift mutations and two in frame insertions/deletions (Fig. 4a). Meanwhile, a total of 53 variations affecting 14 coding sequences (CDS), and 82 variations affecting 37 intron/intergenic regions of strain SyBE_Sc2110M3 were counted (Fig. 4b). Excluding variations in intron/intergenic regions and synonymous substitutions, there was total of 18 sense variations in CDS, including 11 missenses, six frame-shift mutations and one in frame insertions/deletions (Fig. 4b).

**Fig. 5:**
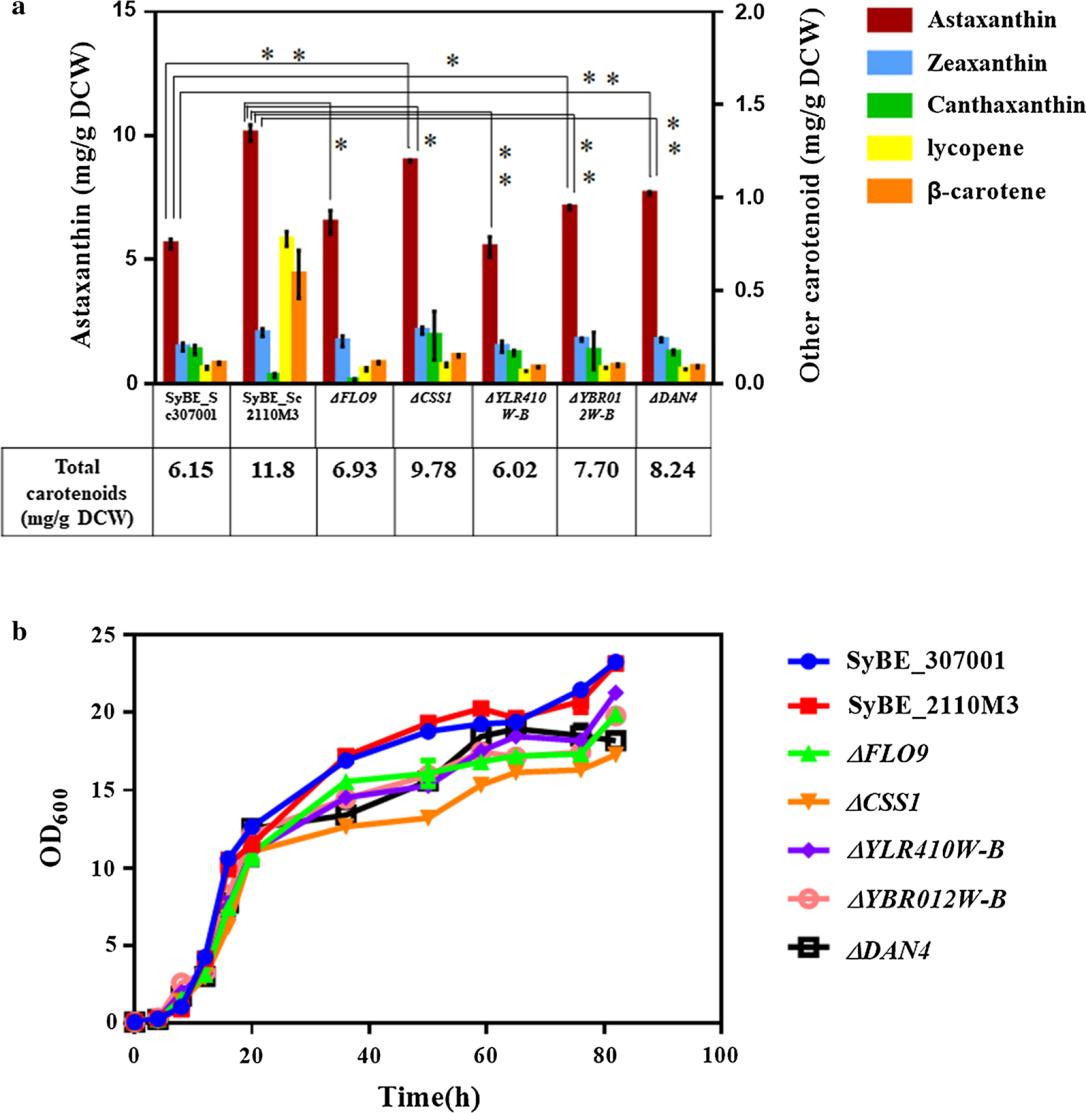
The effect of CDS variations involved in astaxanthin biosynthesis. Carotenoid profile (**a**) and growth curve (**b**) of SyBE_Sc307001, SyBE_Sc2110M3 and gene knocked-out strains in YPD medium. These gene-deleted strains were generated from strain SyBE_Sc307001 by individual loss of genes *FLO9*, *CSS1*, *YLR410W*-*B*, *YBR012W*-*B* and *DAN4*, respectively. “Astaxanthin yield” was determined as “the astaxanthin content in single cell” with unit mg/g DCW. Other carotenoids indicated zeaxanthin, canthaxanthin, lycopene and β-carotene. The error bars represent standard deviation calculated from triplicate experiments. Significance levels of *t*-test were determined as “*” is for *P* < 0.05 and “**” is for *P* < 0.01

Further comparison of CDS variations between SyBE_Sc2110M1 and SyBE_Sc2110M3 only ascribed two variations solely to SyBE_Sc2110M3, i.e. one in *YBR012W*-*B* and one in *YLR410W*-*B* (Table 3). To verify whether their functions were related to astaxanthin yield, these two genes were individually knocked out in the control strain SyBE_Sc307001 (Additional file 1: Figure S6). Based on the annotation by *Saccharomyces* Genome Database (SGD, https://www.yeastgenome.org/) [28], *YBR012W*-*B* and *YLR410W*-*B* happened to be both involved in retrotransposon elements (Table 3). However, only deletion of *YBR012W*-*B* can significantly increase astaxanthin yield, while loss of *YLR410W*-*B* appeared to have no effect on astaxanthin yield (Fig. 5a). Retrotransposon can trigger chromosomal rearrangements and bring global perturbation on transcriptional profiles [29]. So far, it is difficult to interpret the different effect of *YBR012W*-*B* and *YLR410W*-*B* on astaxanthin accumulation. Further transcriptome analysis might provide some clues for related underlying molecular mechanisms in future study.

**Table 3:** CDS variations in SyBE_Sc2110M1 and SyBE_Sc2110M3 with respect to SyBE_Sc307001

Chromosome	Gene	Position and mutate	Function
Specific to SyBE_Sc2110M1			
ChrIV	*HKR1*	1308011: V<->A^a^	Zinc finger family member
		1308047: V<->A^a^	
	*URC2*	1482052: Y<->C^a^	Putative transcription factor
ChrVII	*PDR1*	470224: C<->F^a^	Transcription factor
	*GTO1*	797427: K<->Q^a^	Glutathione transferase
ChrXII	*UBR2*	188380: Q<->H^a^	Cytoplasmic ubiquitin-protein ligase
ChrXIII	*YMR317W*	908196: W<->S^a^	Hypothetical protein
		908198: A<->S^a^	
	*YML084W*	99616D: AT^b^	Hypothetical protein
ChrXV	*NDD1*	1036169D: TTG^c^	Transcriptional activator
	*YOL166C*	1109D: TA^b^	Hypothetical protein
ChrXVI	*RRG8*	765953: N<->K^a^	Hypothetical protein
Specific to SyBE_Sc2110M3			
ChrII	*YBR012W*-*B*	263145D: C^b^	Retrotransposon TYA Gag
ChrXII	*YLR410W*-*B*	944238: Q<->K^a^	Retrotransposon TYA Gag and TYB Pol
Commonalities			
ChrI	*FLO9*	27080: V<->I^a^	Mannose-binding lectin
		25872: I<->M^a^	
		26144: M<->L^a^	
ChrII	*YBL100W*-*A*	30754: D<->N^a^	Retrotransposon TYA Gag
ChrIII	*TAF2*	204436I: CTTCCTCTTCC^b^	RNA polymerase II transcription initiation
ChrIV	*YDR340W*	1150913I: G^b^	Hypothetical ORF
ChrVIII	*YHL050C*	1748: N<->S^a^	Hypothetical ORF
	*YHL041W*	17546I: T^b^	Hypothetical ORF
ChrIX	*CSS1*	25586: D<->G^a^	Hypothetical ORF
		25418: D<->G^a^	
ChrX	*DAN4*	715095: P<->S^a^	Cell wall mannoprotein
		715122: P<->S^a^	
	*YJR023C*	470178: D: ATA^c^	Protein required for cell viability
ChrXI	*YKL225W*	458I: A^b^	Hypothetical ORF
ChrXV	YOR192C-B	708819: A<->G^a^	Retrotransposon TYA Gag
ChrXIV	*YNL338W*	6637D: AC^b^	Hypothetical ORF
ChrXVI	*DPB2*	896899: F<->Y^a^	DNA polymerase epsilon

^a^Missense substitutions, numbers indicated the mutagenesis sites in chromosome, capital letters indicated the corresponding mutations on amino acids

^b^Frame-shift mutations, numbers indicate the mutagenesis sites in chromosome, “D” is for deletion, “I” is for insertion, capital letters after “:” indicates the deleted or inserted base(s)

^c^In frame insertions/deletions, numbers indicate the mutagenesis sites in chromosome, “D” is for deletion, capital letters after “:” indicates the deleted or inserted base(s)

Mutations in SyBE_Sc2110M1 and SyBE_Sc2110M3 can boost total carotenoid accumulation (by more than 0.25-fold) (Fig. 3b). As shown in Table 3, variations in genes *FLO9*, *CSS1* and *DAN4* occurred in high frequency within both SyBE_Sc2110M1 and SyBE_Sc2110M3. Thus, it was assuming that these genes might be crucial to total carotenoid accumulation, and even astaxanthin yield. To support this hypothesis, *FLO9*, *CSS1* and *DAN4* were also individually knocked out in the control strain SyBE_Sc307001 (Additional file 1: Figure S6). As illustrated in Fig. 5a, compared to the control strain, neither astaxanthin yield nor total carotenoid accumulation was significantly enhanced by single deletion of *FLO9*. Nevertheless, compared to the control strain, individual deletion of *CSS1* or *DAN4* enhanced astaxanthin yield by 0.596- and 0.363-fold, respectively (Fig. 5a). Notably, ∆*CSS1* achieved 88.9% of the astaxanthin yield of ARTP-mutant SyBE_Sc2110M3 (Fig. 5a), indicating *CSS1* played a more critical role in astaxanthin biosynthesis than *DAN4*.

**Fig. 6:**
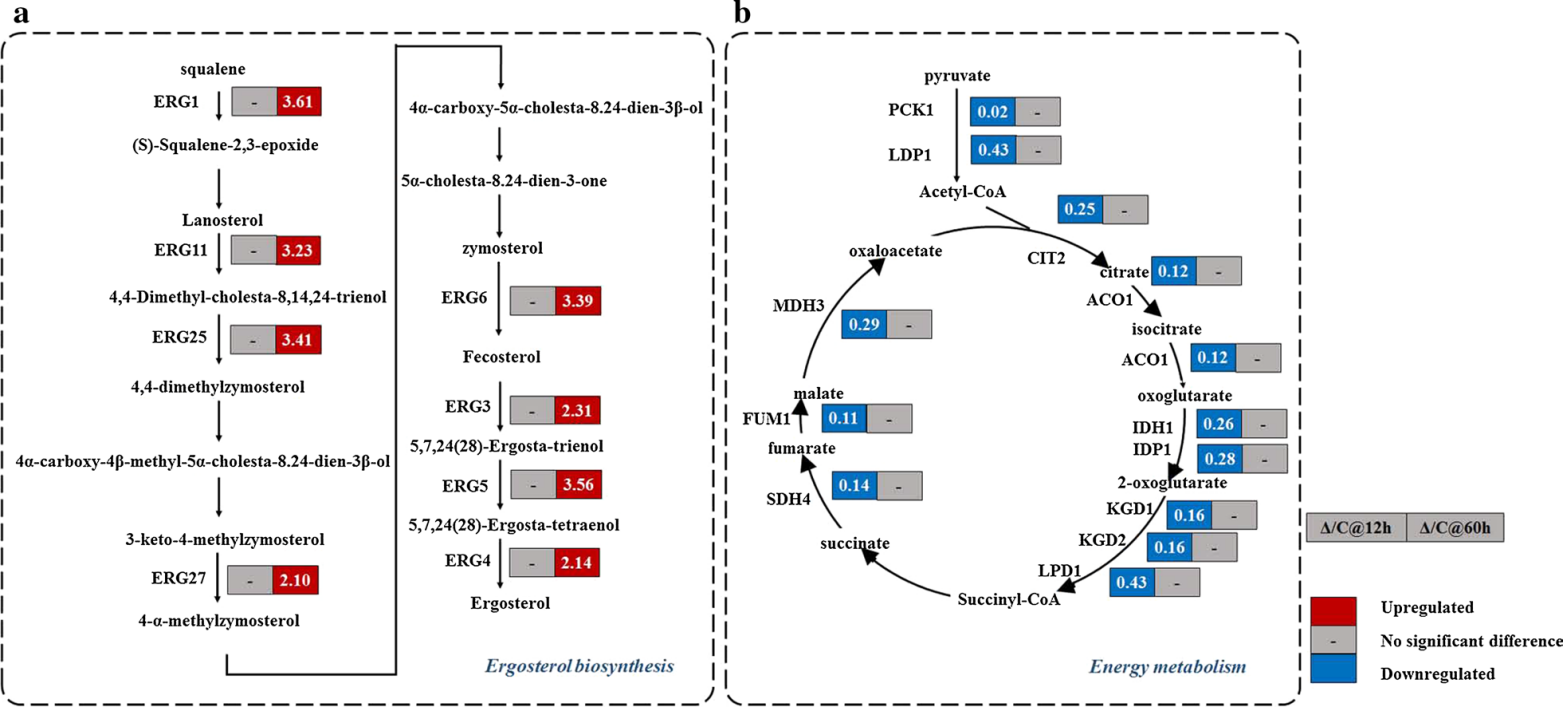
Transcriptional profiles of ergosterol biosynthesis (**a**) and energy metabolism (**b**) by Δ*CSS1*. Δ*CSS1* strain (Δ) and control strain (C) were cultured in YPD medium. Samples were taken at 12 h (early phase) and 60 h (late phase). Number in each box represents transcriptional change (Δ/C), which is the ratio of transcriptional level of Δ to that of C. Upregulated and downregulated genes, and genes without significant transcriptional difference by ΔCSS1 were highlighted in red and blue, and gray, respectively

In SGD, CSS1 is annotated as a putative glucan alpha-1,4-glucosidase. Knockout of its homolog *ISC1* (inositol phosphosphingolipid phospholipase C) in *S. cerevisiae* was found to accumulate more sphingolipids in cell membrane [30]. Consequently, RNA-seq analysis was applied to investigate the transcriptional effect of Δ*CSS1* by comparing the control strain SyBE_Sc307001 with *CSS1* knocked-out strain SyBE_Sc307109. Samples were taken at 12 h (early phase) and 60 h (late phase). As a result, when *CSS1* was lost, genes associated with phospholipid metabolism showed no significant transcriptional difference. However, most of genes involved in biosynthesis of ergosterol (*ERG1*, *ERG11*, *ERG25*, *ERG27*, *ERG6*, *ERG3*, *ERG5 and ERG4*) were found to be upregulated in late phase (Fig. 6a), suggesting Δ*CSS1* might enhance the intracellular sterol levels. Sterols are key components of the cytomembrane and are associated with cell tolerance to hydrophobic molecules (such as d-limonene) [31]. As reported, carotenoids tend to accumulate in membrane system [32], and membrane engineering via altering cell membrane composition has been proved to be a promising strategy to enhance the accumulation of desired hydrophobic products [33, 34]. Therefore, the improvement on astaxanthin yield by Δ*CSS1* is probably due to its biofunction involving in cell membrane. Meanwhile, the RNA-seq data also illustrated that majority of genes involved in TCA cycle (*PCK1*, *LDP1*, *CIT2*, *ACO1*, *IDH1*, *IDP1*, *KGD1/2*, *LPD1*, *SDH4*, *FUM1*, *MDH3*) were found to be downregulated in early phase (Fig. 6b), which might explain the cell growth-deficient problems observed in strains with *∆CSS1* in both YPD medium (Fig. 5b) and SD medium (Additional file 1: Figure S7). As demonstrated in Fig. 5 and Additional file 1: Figure S7, the higher astaxanthin production was caused by the combined effects of gene deletion (such as ∆*CSS1* and *∆YBR012W*-*B*) and reduction in dry cell weight. Since strain SyBE_Sc2110M3, which carried all the gene variations, demonstrated comparable cell growth with the control strain (Fig. 5b and Additional file 1: Figure S7), further gene deletion in different combination such as deletion of *CSS1/DAN4* and *CSS1/FLO9* would be carried to show variations in cell growth.

### Astaxanthin overproduction by fed-batch fermentation

**Fig. 7:**
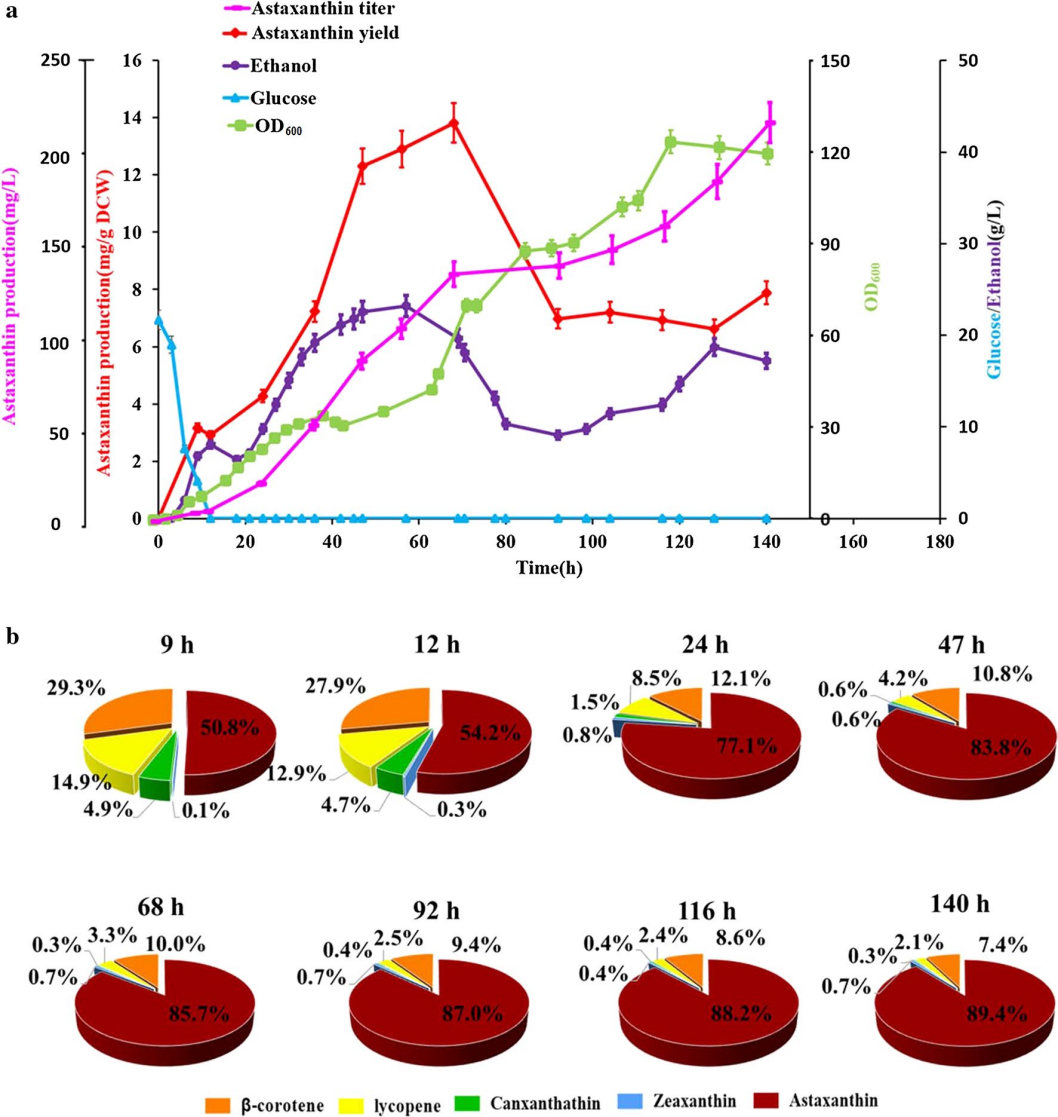
Astaxanthin production in fed-batch fermentation. **a** Profile of astaxanthin yield (pink), astaxanthin titer (red), glucose (blue), ethanol (purple) and cell density (green) during fermentation with strain SyBE_Sc2110M3. The error bars represent standard deviation calculated from duplicate experiments. “astaxanthin yield” was determined as “the astaxanthin content in single cell” with unit mg/g DCW; “astaxanthin titer” was determined as “the astaxanthin concentration in bioreactor” with unit mg/L. **b** The trend of carotenoid composition (the content (mg/g DCW) of a particular carotenoid to the total carotenoid content (mg/g DCW) in single cell, resented in %) in fermentation product. Astaxanthin, zeaxanthin, canthaxanthin, lycopene and β-carotene were labeled by red, blue, green, yellow and orange, respectively

Before bioprocess optimization, the genetic stability of strain SyBE_Sc2110M3 was investigated. As shown in Additional file 1: Figure S8, the astaxanthin yield in strain SyBE_Sc2110M3 was stable after passing six generations in SD medium. Thus, strain SyBE_Sc2110M3 was selected for fed-batch fermentation to further promote the astaxanthin production. To achieve high cell density fermentation for high astaxanthin accumulation, carbon restriction strategy was applied according to Wang et al. [25]. Fed-batch fermentation was conducted in 5-L bioreactor and the glucose feeding was strictly controlled below 2 g/L. Finally, the total volume of the endpoint fermentation in 5-L bioreactor was almost 3.9 L. As shown in Fig. 7a, after the initial glucose was exhausted at 12 h, astaxanthin yield entered a sharply increasing period until the yield reached maximal value (13.8 mg/g DCW) at 68 h. This astaxanthin yield obtained in 5-L bioreactor is higher than that at flask-flash level (10.1 mg/g DCW). In the meantime, the main intermediates (lycopene, β-carotene, canthaxanthin and zeaxanthin) were gradually converted and converged to the targeted product astaxanthin. The intermediate conversion fastigium was observed in the period from 12 to 47 h, and the astaxanthin ratio was sharply enhanced from 54.2 to 83.8% (Fig. 7b). It was also noted that the cell density increased faster than astaxanthin titer after 68 h, leading to a dramatic decrease in astaxanthin yield. Such threshold indicated we should optimize the feeding strategies to balance the cell growth and target product production in future work. Eventually, a titer of 217.9 mg/L astaxanthin was obtained after 140 h cultivation (Fig. 7a). At that point, the astaxanthin ratio reached to 89.4% (Fig. 7b). Indeed, the titer was not satisfactory for large-scale commercialization. Considering the cell stress caused by astaxanthin accumulation [35], exploring inducible promoters (such as GAL promoters) to control the expression of heterologous genes could achieve high cell-density fermentation by decoupling cell growth with product accumulation, which could significantly shorten the cell growth period and concentrate optimization for astaxanthin yield enhancement by corresponding feeding strategy. To figure out potential biomarkers by comparing different astaxanthin accumulation period by metabolomics analysis could also be a powerful source to guide media and feeding strategy optimization [36].

## Conclusions

A comprehensive approach integrating heterologous module engineering and mutagenesis by ARTP was employed to promote astaxanthin yield to 10.1 mg/g DCW, which is the highest reported yield at shake-flask level in *S. cerevisiae* so far. Astaxanthin yield and ratio was significantly enhanced by involving new combination of CrtZ and CrtW (Aa_CrtZ–BDC263_CrtW). Three underlying gene targets (*CSS1*, *YBR012W*-*B* and *DAN4*) associated with astaxanthin biosynthesis were first uncovered by comparative genomics analysis. Notably, deletion of *CSS1* can recover 75.6% improvement on astaxanthin yield by ARTP, indicating *CSS1* is a key molecular target for astaxanthin accumulation. RNA-seq analysis indicates that CSS1 deletion effectively upregulates membrane composition synthesis. Eventually, 217.9 mg/L astaxanthin (astaxanthin ratio was 89.4% and astaxanthin yield was up to 13.8 mg/g DCW) was obtained in 5-L fermentor without any addition of inducers. This combinatorial strategy might be also applicable for biosynthesis of other value-added products, especially colored metabolites. And either increasing the round of mutagenesis or iterative operation of metabolic engineering and mutagenesis by screening in several rounds would be very promising for future optimization. In terms of current system, exploring process optimization (i.e. medium optimization and feeding strategy testing) to balance the carbon flux towards biomass built-up and astaxanthin biosynthesis is also very helpful for further improvement, in which more on-line parameter feedback control strategy guided by off-gas analysis will be very essential [37].

## Methods

### Strains and cultivation

All the strains used in this study are described in Table 2. *E. coli* DH5α was used for plasmid construction and replication. *E. coli* strains were cultured at 37 °C in Luria–Bertani (LB) complete medium. 50 µg/mL kanamycin or 100 µg/mL ampicillin was added into the medium for selection. In the meanwhile, yeast cells were cultured at 30 °C in YPD medium [27]. For astaxanthin fermentation in shake-flask, a single yeast colony was inoculated into 3 mL SD medium [27] and grown at 30 °C until OD_600_ ≈ 8.0. Then the preculture was inoculated into 3 mL fresh SD medium with an initial OD_600_ of 0.2 for further 14 h cultivation (to OD_600_ ≈ 6.0). After that, the seed culture was transferred into 50 mL fresh YPD medium for 84 h at an initial OD_600_ of 0.1 and grew until harvest.

### Construction of plasmids and strains

All the plasmids used in this study are listed in Additional file 1: Table S1. Genes encoding CrtW from *B. vesicularis* DC263 (BDC263_CrtW) and CrtZ from *A. aurantiacum* (Aa_CrtZ) were recovered by BsaI digestion from plasmids pUC57-Simple-14 and pUC57-Simple-01, respectively. These two plasmids were generated in our previous work via cloning genes *BDC263_crtW* and *Aa_crtZ* (codon optimized and synthesized by Genscript Inc.) into plasmid pUC57-simple [25]. Construction of BDC263_CrtW–Aa_CrtZ expression cassette plasmid (pRS425K-BDC263_CrtW–Aa_CrtZ) (Additional file 1: Figure S1a) and CrtZ expression cassette plasmid (pRS425K-Asp_CrtZ/pRS425K-Aa_CrtZ) (Additional file 1: Figure S2a) followed the procedure described in our previous work [25]. The constructed product was verified by *Pst*I/*Bam*HI digestion and DNA-sequencing. Transformation of the particular plasmid into strain SyBE_Sc118030 was conducted by LiAc/SS carrier DNA/PEG method [38]. The engineered yeast strains were selected on SD medium with the appropriate amino acid drop out mix supplementation.

PCR-mediated gene disruptions were applied with the primers provided in Additional file 1: Table S2. Yeast homologous arms and *KanMX* marker were amplified from the genomic DNA of *S. cerevisiae* SyBE_Sc118030 and plasmid bYW0182, respectively. The gene knockout cassette (left homologous arm-*KanMX*-right homologous arm) was assembled by overlap extension PCR (OE-PCR). Then the product was purified and transformed into strain SyBE_Sc307001. The gene-deleted strains were verified by PCR with the primers listed in Additional file 1: Table S2. One pair of the verified primers (I-F/I-R, Additional file 1: Figure S6a) was located upstream of the left homologous arm and downstream of the right homologous arm of the targeted gene; while another pair of the verified primers (II-F/II-R, Additional file 1: Figure S6a) was located inside of the targeted gene. Primers I-F/I-R are able to amplify a clear band in the DNA poles for gene knocked-out stains (i.e. 2960 bp for *∆FLO9*, 2089 bp for *∆CSS1*, 2296 bp for *∆DAN4*, 3277 bp *∆YBR012W*-*B,* 2745 bp for *∆YLR410W*-*B*) while primers II-F/II-R are able to amplify a clear band only in the DNA poles for the parent stain (i.e. 2012 bp for *FLO9*, 1823 bp for *CSS1*, 2168 bp for *DAN4*, 2054 bp for *YBR012W*-*B*, 1966 bp for *YLR410W*-*B*).

### Construction of ARTP mutagenesis library

After growing in SD medium for 30 h, the culture was transferred to fresh SD medium until OD_600_ approaches 1.0. Then 10 μL of the culture was spread on a sterile iron plate to be irradiated by ARTP with a power of 100 W and a gas flow of 10 SLM. The distance between the plasma torch nozzle exit and the sample plate was 2 mm. Since it requires a death rate of 80%–90% to achieve effective mutation [39, 40], cells were submitted to ARTP for 10, 15, 20, 30, 40, 50, 60, 80, 100, 120 and 150 s, respectively. After that, all the treated cells were washed from the plate and further grown on SD agar plates for 48 h. It was found that only 30-s and 40-s treatments could obtain a death rate between 80 and 90% (i.e. 82.6% for 30 s and 86.8% for 40 s, Additional file 1: Figure S3). Thus, our ARTP mutagenesis library covered the cells treated by ARTP for both 30 s and 40 s. After obtaining ARTP mutagenesis library, visual color screening [25] could be performed to choose the candidates with dark red color for higher astaxanthin yield. The genetic stability assay was conducted in SD medium. The candidate strain(s) was sub-cultured for six generations. And the astaxanthin yield was monitored accordingly.

### Quantitative real-time PCR

Quantitative real-time PCR (qPCR) was applied to quantify copy numbers of plasmid or to measure the transcriptional levels of genes in engineered strains. The copy number assay was referred to Jia et al. [41]. Yeast genomic DNA was employed as the template in qPCR analysis. Strain SyBE_Sc307120 with single copy *LEU2* marker was used as the reference strain. The copy numbers were determined by comparing the Ct values of *LEU2* and the reference gene *ALG9* using the 2^−ΔΔCt^ method [42]. For transcription level analysis, strains were sampled at (early phase) and 60 h (late phase), respectively. The method of total RNA extraction, reverse transcription and qPCR procedure was as same as Wang et al. [25], except gene ALG9 was adopted for normalization. The relative transcription level for CrtZ and CrtW was determined as − ΔCt [43], using gene ALG9 for normalization. The relative ratio of CrtZ to CrtW was calculated as 2^−ΔCt(CrtZ)^/2^−ΔCt(CrtW)^.

### Genome re-sequencing and comparative genome analysis

Whole genome re-sequencing of strains SyBE_Sc307001, SyBE_Sc2110M1 and SyBE_Sc2110M3 was carried out by Beijing Novogene Bioinformatics Technology Co., Ltd. Yeast cultures were grown to saturation, and then genomic DNA was isolated to prepare amplicon-free libraries. Paired-end sequencing for all libraries was performed on the Illumina HiSeq2000 platform. Burrows–Wheeler alignment (BWA) [44] was used to align the paired-end reads against the sequences of *S. cerevisiae* S288C (Accession No. NC_001136) and the heterologous modules provided by our group. SAMtools [45] was used to detect the individual SNPs (single nucleotide polymorphisms) and Indels (insertion and deletion of fragments < 50 bp), as well as analyze the variation of SNPs/InDels between strains SyBE_Sc2110M1/M3 and SyBE_Sc307001.

#### RNA-sequencing analysis

Cells were harvested from cultivation at 12 h (early phase) and 60 h (late phase), respectively. Total RNA was isolated following the NEB Next Ultra™ RNA protocol and using NEB Next Poly(A) mRNA Magnetic Isolation Module (NEB) according to the manufacturer’s instructions and was quantified and qualified by Agilent 2100 Bioanalyzer. Sequencing was processed by Genewiz Inc. on Illumina HiSeq2500 platform. Image analysis and base calling were conducted by HiSeq control software on the HiSeq instrument. Htseq software was used to normalize the data using RPKM (Reads per Kilo bases per Million reads)-based normalization algorithm [46]. Differentially expressed genes were identified by DESeq2 software [47] with log2foldchange > 1.0 and a corrected *P*-value < 0.05. R software was used for hierarchical clustering analysis. RNA-seq data were calculated from two biological replicates. The *Saccharomyces* genome database (SGD) [28] was used to gain gene information.

### Fed-batch fermentation

Fed-batch fermentation was conducted in a 5-L bioreactor (T&J Bioengineering Co., Ltd, Shanghai, China) under glucose restriction strategy. 100 µL glycerol-stock of strain SyBE_Sc2110M3 was inoculated into 50 mL SD medium and cultured at 30 °C, 250 rpm for overnight growth. Then the preculture was transferred to 200 mL fresh SD medium and grew until entering mid-exponential phase. 10% (v/v) seed culture was transferred to 1.8 L YPD medium (containing 20 g/L glucose) in 5-L bioreactor. The fermentation was carried out at 30 °C. The pH was automatically controlled at 5.8 with 6 M sodium hydroxide. And the dissolved oxygen was kept at 30% by agitation cascade from 400 to 800 rpm, while the air flow was set at 1.5 vvm. The glucose concentration was monitored every 3 h. And after the initial glucose was depleted, an appropriate volume of 500 g/L glucose solution was supplemented to maintain the glucose concentration less than 2 g/L. 30 g yeast extract was added into the bioreactor every 12 h by adding 500 g/L yeast extract stock solution. Duplicate samples were collected to determine the cell density, glucose concentration, ethanol concentration and astaxanthin production.

### Determination of glucose, ethanol and carotenoids

The concentrations of glucose and ethanol in the culture were analyzed by a reverse-phase high-performance liquid chromatography (HPLC) system consisting of a Waters 1515 pump (Milford, MA, USA) and a Waters 2414 refractive index detector. The samples were separated on an Aminex HPX-87H carbohydrate analysis column (Bio-Rad, Hercules, CA, USA) at 65 °C using 5 mM sulfuric acid as the mobile phase with a flow rate of 0.6 mL/min.

Carotenoids were extracted from the HCl–heat-treated cells with acetone according to Zhou et al. [11] and Wang et al. [25]. To be specific, cells from 2 mL culture were collected and washed with distilled water. After that, the cells were suspended in 1 mL 3 M HCl for 5 min boiling at 100 °C in sealed tubes and then quickly chilling on ice. The cell debris were harvested, washed with distilled water twice and resuspended in 0.5 mL of acetone containing 1% (w/v) butylated hydroxytoluene. The above mixture was vortexed for 20 min and incubated at 30 °C for 10 min. After centrifugation at 12,000 rpm for 5 min, the acetone phase was filtered by 0.22-μm membrane. The whole process was performed in darkness. And all centrifugal tubes used for astaxanthin extraction were covered with aluminum foil to avoid exposure to light. The extracted products were analyzed by HPLC (Waterse2695, Waters Corp., USA) equipped with a BDS HYPERSIL C18 column (150 mm × 4.6 mm, 5 μm, Thermo Scientific) and a UV/VIS detector (Waters 2489) at 470 nm [25]. Carotenoids standards (astaxanthin, zeaxanthin, canthaxanthin, lycopene and β-carotene) were purchased from Sigma (Sigma-Aldrich, MO, USA) for qualitative and quantitative analysis. To describe astaxanthin productivity, “the astaxanthin content in single cell” was determined as “astaxanthin yield” with unit mg/g DCW; “the astaxanthin concentration in bioreactor” was determined as “astaxanthin titer” with unit mg/L; “the astaxanthin content (mg/g DCW) to the total carotenoids content (mg/g DCW) in single cell” was determined as “astaxanthin ratio” in %.

### Statistical analysis

All the statistical analysis was conducted by SPSS 19.0. The level of significance was set at *P* < 0.05. All the error bars represented at least independent duplicate experiments.

## Additional file

**Additional file 1: Table S1.** Plasmids used in this study. **Table S2.** Primers used in this study. **Figure S1.** The effect of new combination of CrtZ-CrtW on carotenoids profile. (**a**) Sketch map of CrtW-CrtZ expression cassette plasmids. CrtW-CrtZ expression cassette was carried by a multiple copy plasmid pRS425K. Expression modules for CrtW (TDH3p-*crtW*-TDH2t) and CrtZ (FBA1p-*crtZ*-ADH1t) were arranged back-to-back with opposite transcriptional direction. Promoters, encoding sequences and terminators were presented as green, yellow and red, respectively. (**b**) The HPLC profile of strain SyBE_Sc118060 (blue) with BDC263_CrtW-Asp_CrtZ and SyBE_Sc307001 (red) with BDC263_CrtW-Aa_CrtZ. The black line indicated the profiles for mix-standard. Astaxanthin, zeaxanthin, canthaxanthin, lycopene and β-carotene were labeled as I, II, III, IV and V. **Figure S2.** The effect of CrtZ source on zeaxanthin yield. (**a**) Sketch map of CrtZ expression cassette plasmids. Promoters, encoding sequences and terminators were presented as green, yellow and red, respectively. (**b**) Zeaxanthin yield of strain SyBE_Sc307121 and SyBE_Sc307122. These two strains were generated from the same β-carotene producer SyBE_Sc118030 by individually expression of Aa_CrtZ or Asp_CrtZ. **Figure S3.** Effect of various plasma treatment times on the survival rate of SyBE_Sc307001. Data were pooled from three independent experiments. **Figure S4.** Cell growth and astaxanthin yield of ARTP mutants. Growth curve (**a**) and carotenoids profile (**b**) of strain SyBE_Sc307001 and its ARTP mutagenesis strains (SyBE_Sc2110M2, M4-M7) which did not achieve higher total carotenoids accumulation in YPD medium than the parent strain. A photograph was attached bellow the bar chart to illustrate visual color of the related strains. The error bars represent standard deviations calculated from duplicate experiments. The error bars represent standard deviations calculated from duplicate experiments. “Astaxanthin yield” was determined as “the astaxanthin content in single cell” with unit mg/g DCW. **Figure S5.** The copy numbers of plasmid pRS425K-BDC263_CrtW-Aa_CrtZ in strain SyBE_Sc307001, SyBE_Sc3070M1 and SyBE_Sc3070M3. **Figure S6.** PCR verification of the desired gene-deleted strains. (**a**) Sketch map of the design of PCR verify primers. One pair of the verified primers (I-F/I-R) were located upstream of the left homologous arm and downstream of the right homologous arm of the targeted gene; while another pair of the verified primers (II-F/II-R) were located inside of the targeted gene. Primers I-F/I-R are able to amplify a clear band in the DNA poles for gene knocked-out stains; while Primers II-F/II-R are able to amplify a clear band only in the DNA poles for the parent stain. (**b**) Electrophoretic map of PCR products. To be notably, the primers I-F/I-R could amplify the band from control, which should be 5456 bp (for *FLO9*), 4988 bp (for *CSS1*), 4659 bp (for *DAN4*), 6724 bp (for *YBR012W-B*) and 6154 bp (*YLR410W-B*). However, the current extension time was just enough to amplify the band from the gene deleted strains but it was too short (or it was hard) to obtain the band from control. **Figure S7.** Growth curve of strains SyBE_Sc307001, SyBE_Sc2110M3 and gene deleted strains in SC medium. These gene knocked-out strains were generated from strain SyBE_Sc307001 by individual deletion of gene *FLO9, CSS1, YLR410W-B, YBR012W-B and DAN4*, respectively. **Figure S8.** Astaxanthin yield of stain SyBE_Sc2110M3 in each generation cultivated in SD medium.

## Abbreviations

CrtZ: β-carotene hydroxylase
CrtW: β-carotene ketolase
Aa: *Agrobacterium aurantiacum*
Asp: *Alcaligenes* sp. strain PC-1
BDC263: *Brevundimonas vesicularis* DC263
ARTP: atmospheric and room temperature plasma
