# Supplementary material for: Astaxanthin overproduction in yeast by strain engineering and new gene target uncovering
Source: Biotechnol Biofuels. 2018 Aug 23;11:230. doi: 10.1186/s13068-018-1227-4 (PMC6106823; doi:10.1186/s13068-018-1227-4)
Supplement: Supplementary file 1 — Additional file 1: Table S1. Plasmids used in this study. Table S2. Primers used in this study. Figure S1. The effect of new combination of CrtZ-CrtW on carotenoids profile. (a) Sketch map of CrtW-CrtZ expression cassette plasmids. CrtW-CrtZ expression cassette was carried by a multiple copy plasmid pRS425K. Expression modules for CrtW (TDH3p-crtW-TDH2t) and CrtZ (FBA1p-crtZ-ADH1t) were arranged back-to-back with opposite transcriptional direction. Promoters, encoding sequences and terminators were presented as green, yellow and red, respectively. (b) The HPLC profile of strain SyBE_Sc118060 (blue) with BDC263_CrtW-Asp_CrtZ and SyBE_Sc307001 (red) with BDC263_CrtW-Aa_CrtZ. The black line indicated the profiles for mix-standard. Astaxanthin, zeaxanthin, canthaxanthin, lycopene and β-carotene were labeled as I, II, III, IV and V. Figure S2. The effect of CrtZ source on zeaxanthin yield. (a) Sketch map of CrtZ expression cassette plasmids. Promoters, encoding sequences and terminators were presented as green, yellow and red, respectively. (b) Zeaxanthin yield of strain SyBE_Sc307121 and SyBE_Sc307122. These two strains were generated from the same β-carotene producer SyBE_Sc118030 by individually expression of Aa_CrtZ or Asp_CrtZ. Figure S3. Effect of various plasma treatment times on the survival rate of SyBE_Sc307001. Data were pooled from three independent experiments. Figure S4. Cell growth and astaxanthin yield of ARTP mutants. Growth curve (a) and carotenoids profile (b) of strain SyBE_Sc307001 and its ARTP mutagenesis strains (SyBE_Sc2110M2, M4-M7) which did not achieve higher total carotenoids accumulation in YPD medium than the parent strain. A photograph was attached bellow the bar chart to illustrate visual color of the related strains. The error bars represent standard deviations calculated from duplicate experiments. The error bars represent standard deviations calculated from duplicate experiments. “Astaxanthin yield” was determined as “the astaxanthin co [file 13068_2018_1227_MOESM1_ESM.docx]

**Additional information**

**Astaxanthin overproduction in yeast by strain engineering and new gene targets uncovering**

Jin Jin ^a,b#^, Ying Wang^a,b#^, Mingdong Yao^a,b^, Xiaoli Gu^a,b^, Bo Li^a,b^, Hong Liu^a,b^, Mingzhu Ding^a,b^, Wenhai Xiao^a,b^*, Yingjin Yuan^a,b^

^a^ Key Laboratory of Systems Bioengineering (Ministry of Education), Tianjin University, Tianjin, 300072, P.R. China.

^b^ SynBio Research Platform, Collaborative Innovation Center of Chemical Science and Engineering (Tianjin), Tianjin University, Tianjin, 300072, P.R. China.

*Corresponding author:

Wenhai Xiao (Email: wenhai.xiao@tju.edu.cn, Tel: 86-22-60973987, Postal address: No. 92, Weijin Road, Nankai District, Tianjin, 300072, PR China)

**Table S1.** Plasmids used in this study

| **Plasmid** | **Description** | **Sources** |
| --- | --- | --- |
| bYW0182 | Plasmid providing *KanMX* marker | This study |
| pUC57-Simple | Blunt Cloning vector, resistant to ampicillin | GenScript |
| pUC57-Simple-01 | Plasmid harboring gene *crtZ* from *Agrobacterium aurantiacum* (*Aa_crtZ*) | [1] |
| pUC57-Simple-14 | Plasmid harboring gene *crtW* from *Brevundimonas sp. DC263* (*BDC263_crtW*) | [1] |
| pRS425K- Asp_CrtZ | Multiple copies plasmid harboring Asp_CrtZ expression cassette (FBA1p-*Asp_crtZ*-ADH1t) | This study |
| pRS425K- Aa_CrtZ | Multiple copies plasmid harboring Aa_CrtZ expression cassette (FBA1p-*Aa_crtZ*-ADH1t ) | This study |
| pRS425K-BDC263_CrtW-Aa_CrtZ | Multiple copies plasmid harboring BDC263_CrtW-Aa_CrtZ expression cassette (ADH1t-*Aa_crtZ*-FBA1p-TDH3p-*BDC263_crtW*-TDH2t) | This study |

**Reference**

1. Wang R, Gu X, Yao M, Pan C, Liu H, Xiao W, Wang Y, Yuan Y. Engineering of β-carotene hydroxylase and ketolase for astaxanthin overproduction in Saccharomyces cerevisiae. Front Chem Sci Eng. 2017;11:89-99.

**Table S2.** Primers used in this study

| **Primer** | **Sequence (5’- 3’)** | |
| --- | --- | --- |
| **For deletion of** ***FLO9*** | | |
| FLO9_LF | | AAGCGAACCACACTAGATCTTACG |
| FLO9_LR | | TATTCTGGGCCTCCATGTCCTTACTGGCCGGTAGTGGTTTAAG |
| KanMX _F | | AACCACTACCGGCCAGTAAGGACATGGAGGCCCAGAATAC |
| KanMX _R | | TCGTCACATTGCTGGGATTAACCAGTATAGCGACCAGCATTC |
| FLO9_RF | | AATGCTGGTCGCTATACTGGTTAATCCCAGCAATGTGACGATGG |
| FLO9_RR | | AGCGATGGGTACTTTCCTTCATAGG |
| **PCR verification of *△FLO9*** | | |
| FLO9_VF1 | | TACTGCGTATGGCATGCACAG |
| FLO9_VR1 | | TCGAGATCAGAGGTCACATAGG |
| FLO9_VF2 | | GACGTGATAAAGCTGGTGAT |
| FLO9_VR2 | | GGGACTGTCTACATGTATGCTG |
| **For deletion of *CSS1*** | | |
| CSS1_LF | | ACATATCGGAAGCCCACAAATAACTGCAAC |
| CSS1_LR | | TTGCTGAGACATCCTCGTCGACATGGAGGCCCAGAATA |
| KanMX _F | | TATTCTGGGCCTCCATGTCGACGAGGATGTCTCAGCAA |
| KanMX _R | | AATGCTGGTCGCTATACTGTACTGTCACTTCTGAGGGTT |
| CSS1_RF | | AACCCTCAGAAGTGACAGTACAGTATAGCGACCAGCATT |
| CSS1_RR | | GGCAGAAGAGGGAACCATTTCATCCAA |
| **PCR verification of *△CSS1*** | | |
| CSS1_VF1 | | AGTCCAAACGGCTCATTTTG |
| CSS1_VR1 | | ATAAAGCCAAGTGCTCAGAA |
| CSS1_VF2 | | CGGTAACTTCAACGCTGAAG |
| CSS1_VR2 | | CCCTCAGAAGTGACAGTAGTG |
| **For deletion of** ***YLR410W-B*** | | |
| YLR410W-B_LF | | CGCCTATGCTTCGGTTACTTCTAAGGAAGT |
| YLR410W-B _LR | | ACTATCCGTTGCCACAGTATGACATGGAGGCCCAGAATAC |
| KanMX _F | | GTATTCTGGGCCTCCATGTCATACTGTGGCAACGGATAGT |
| KanMX _R | | GAATGCTGGTCGCTATACTGGCACAAAGGCAATGAGACTT |
| YLR410W-B _RF | | AAGTCTCATTGCCTTTGTGCCAGTATAGCGACCAGCATTC |
| YLR410W-B _RR | | TCGGAGAAGTGAAGAGAATGTGGATTTTGA |
| **PCR verification of *△YLR410W-B*** | | |
| YLR410W-B _VF1 | | TAGCGCCATAAGGATATGCGT |
| YLR410W-B _VR1 | | GCTGCTACTGCCTCCCATAT |
| YLR410W-B _VF2 | | CCTTCGCACATTTCAAAGCT |
| YLR410W-B _VR2 | | ATCTTGGTGGTTCCAGACTT |
| **For deletion of** ***YBR012W-B*** | | |
| YBR012W-B_LF | | CGCCTGTGCTTCGGTTACTTCTAAGGAAGT |
| YBR012W-B _LR | | TCACAGTTTCCGCAGTATCCGACATGGAGGCCCAGAATAC |
| KanMX _F | | GTATTCTGGGCCTCCATGTCGGATACTGCGGAAACTGTGA |
| KanMX _R | | GAATGCTGGTCGCTATACTGAGACGTAATGACCAAACCTC |
| YBR012W-B _RF | | GAGGTTTGGTCATTACGTCTCAGTATAGCGACCAGCATTC |
| YBR012W-B _RR | | AGGCCTTTGATAATGCCCTTGAAAATTCAC |
| **PCR verification of *△YBR012W-B*** | | |
| YBR012W-B _VF1 | | TTCCACTTCGTTGAAGCCTCAC |
| YBR012W-B _VR1 | | CAGCTACACCAGGACAGCAT |
| YBR012W-B _VF2 | | CGACTGGTCTAGTGCTATTGAC |
| YBR012W-B _VR2 | | GATGCTGAATATCACCTCTTGC |
| **For deletion of** ***YDR544C*** | | |
| YDR544C_LF | | ACCATGATAGTGTTGCTACCACAAG |
| YDR544C _LR | | TATTCTGGGCCTCCATGTCCCACATGCCATACTCACCTTC |
| KanMX _F | | AAGGTGAGTATGGCATGTGGGACATGGAGGCCCAGAATAC |
| KanMX _R | | ATGTCCCTACGGCCTTGTCTCAGTATAGCGACCAGCATTC |
| YDR544C _RF | | AATGCTGGTCGCTATACTGAGACAAGGCCGTAGGGACATATAG |
| YDR544C _RR | | CTGCGCTTAGCCTACAACTTCTTC |
| **PCR verification of *△YDR544C*** | | |
| YDR544C _VF | | ATCCGTTTAAGTATACGCCGCT |
| YDR544C _VR | | TCTTCGTAGAATGACCGCACT |
| **For deletion of** ***DAN4*** | | |
| DAN4_LF | | GTACCAGTTCCTGTCGAATCTGCGATATCC |
| DAN4_LR | | TTTACACGGCTATCCCAACCGACATGGAGGCCCAGAATAC |
| KanMX _F | | GTATTCTGGGCCTCCATGTCGGTTGGGATAGCCGTGTAA |
| KanMX _R | | AATGCTGGTCGCTATACTGCTACTGTCGATGCAGGGATT |
| DAN4_RF | | AATCCCTGCATCGACAGTAGCAGTATAGCGACCAGCATT |
| DAN4_RR | | ACTTGTTGGCGATACCTTAGAGGATTCAA |
| **PCR verification of *△DAN4*** | | |
| DAN4_VF1 | | ACGAGCTAAGATCCTCTGAA |
| DAN4_VR1 | | AGAATTTAGCAGCGTCAACT |
| DAN4_VF2 | | GTACCAGTTCCTGTCGAATCT |
| DAN4_VR2 | | GGCTGAAGTGAAAGTGGATG |
| **For qPCR** | |  |
| act1-F | | CGTTCCAATTTACGCTGGTT |
| act1-R | | GGCCAAATCGATTCTCAAAA |
| crtW-F | | AGCGTGATGAGCGTGATGG |
| crtW-R | | ACTTGGTTGTCCGTAGGTTTATTC |
| AspcrtZ-F | | CTGTCTTGTGGTGGATTGC |
| AspcrtZ-R | | CTTCTAGGTATGTATCTGAATGGC |
| AacrtZ-F | | TGTTCACTGTTGGTTGGATC |
| AacrtZ-R | | GCCATCTTTGGTGAACCAA |
| Leu-F | | CTATGTCTGCCCCTAAGAAG |
| Leu-R | | GGAACGAACATCAGAAATAGC |

**a**

**
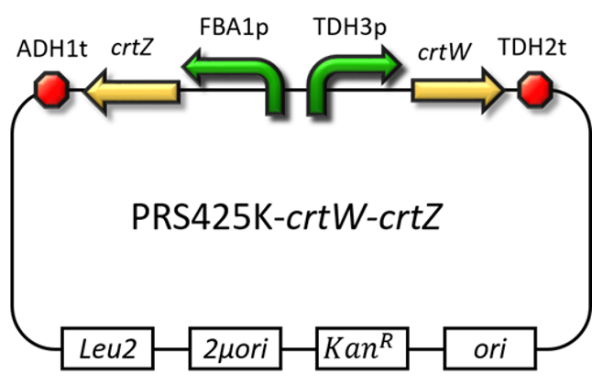
**

**b**

**
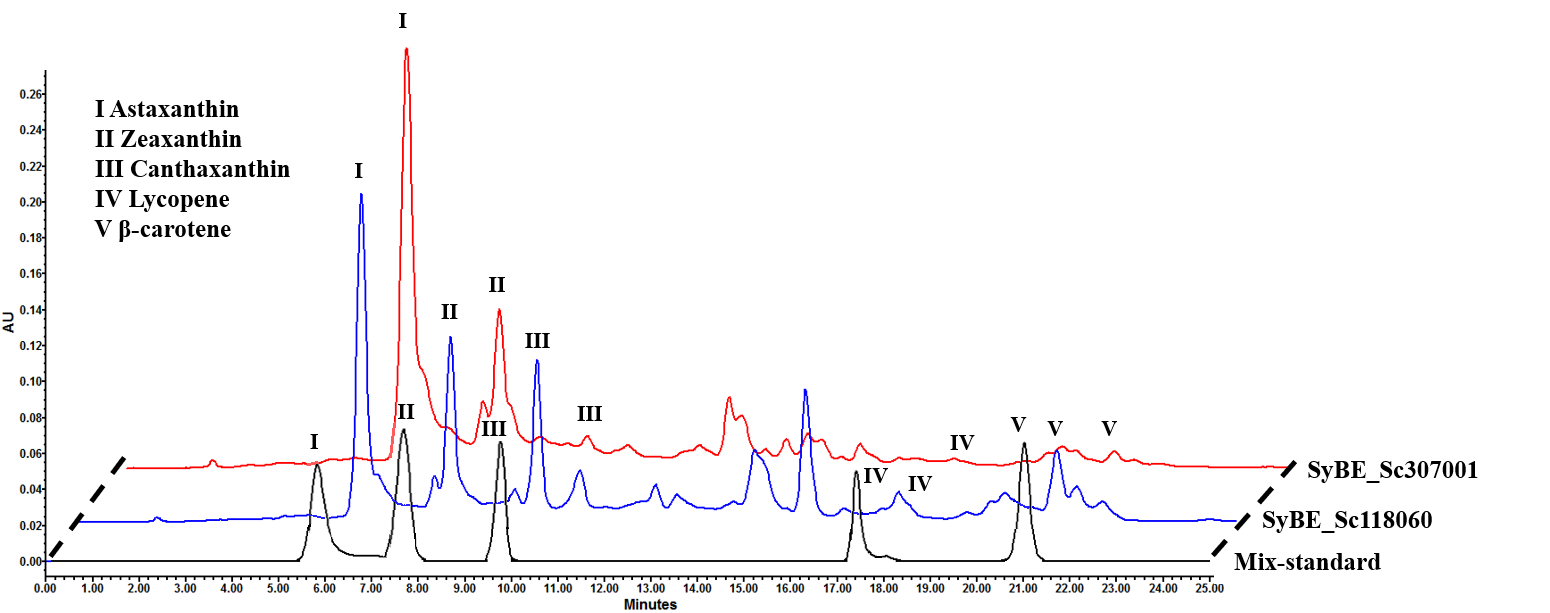
**

**Figure S1.** The effect of new combination of CrtZ-CrtW on carotenoids profile. (**a**)

Sketch map of CrtW-CrtZ expression cassette plasmids. CrtW-CrtZ expression cassette was carried by a multiple copy plasmid pRS425K. Expression modules for CrtW (TDH3p-*crtW*-TDH2t) and CrtZ (FBA1p-*crtZ*-ADH1t) were arranged back-to-back with opposite transcriptional direction. Promoters, encoding sequences and terminators were presented as green, yellow and red, respectively. (**b**) The HPLC profile of strain SyBE_Sc118060 (blue) with BDC263_CrtW-Asp_CrtZ and SyBE_Sc307001 (red) with BDC263_CrtW-Aa_CrtZ. The black line indicated the profiles for mix-standard. Astaxanthin, zeaxanthin, canthaxanthin, lycopene and β-carotene were labeled as I, II, III, IV and V.

**
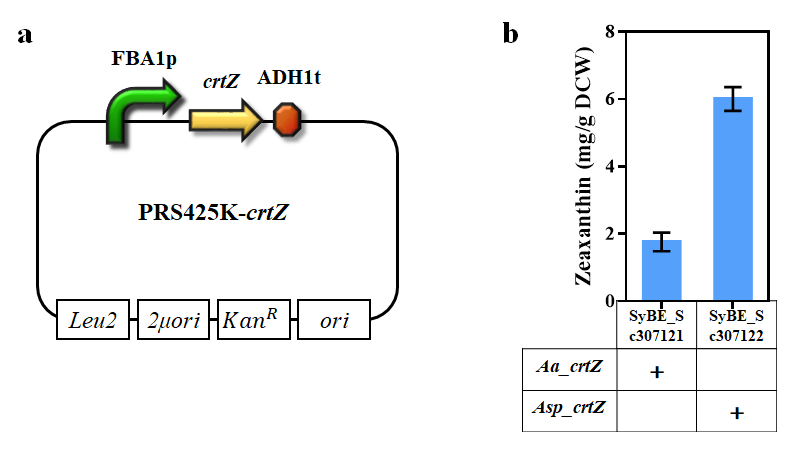
**

**Figure S2.** The effect of CrtZ source on zeaxanthin yield. (**a**) Sketch map of CrtZ expression cassette plasmids. Promoters, encoding sequences and terminators were presented as green, yellow and red, respectively. (**b**) Zeaxanthin yield of strain SyBE_Sc307121 and SyBE_Sc307122. These two strains were generated from the same β-carotene producer SyBE_Sc118030 by individually expression of Aa_CrtZ or Asp_CrtZ.

**
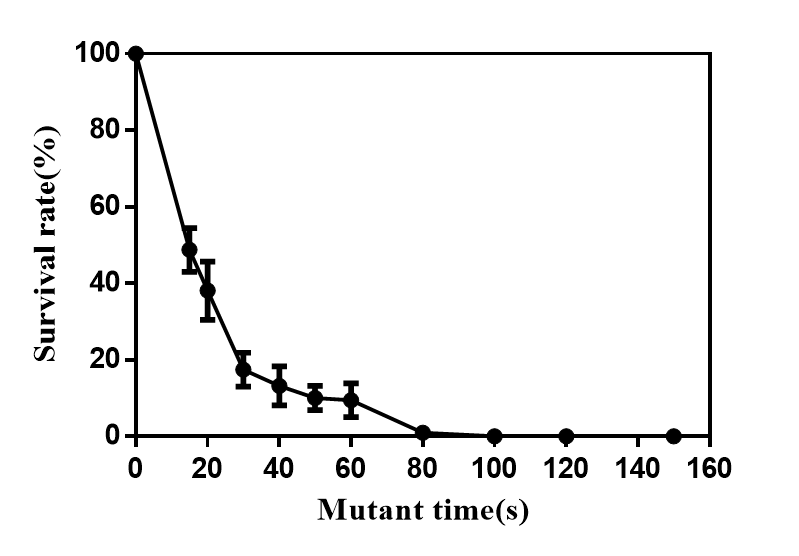
**

**Figure S3.** Effect of various plasma treatment times on the survival rate of SyBE_Sc307001. Data were pooled from three independent experiments.


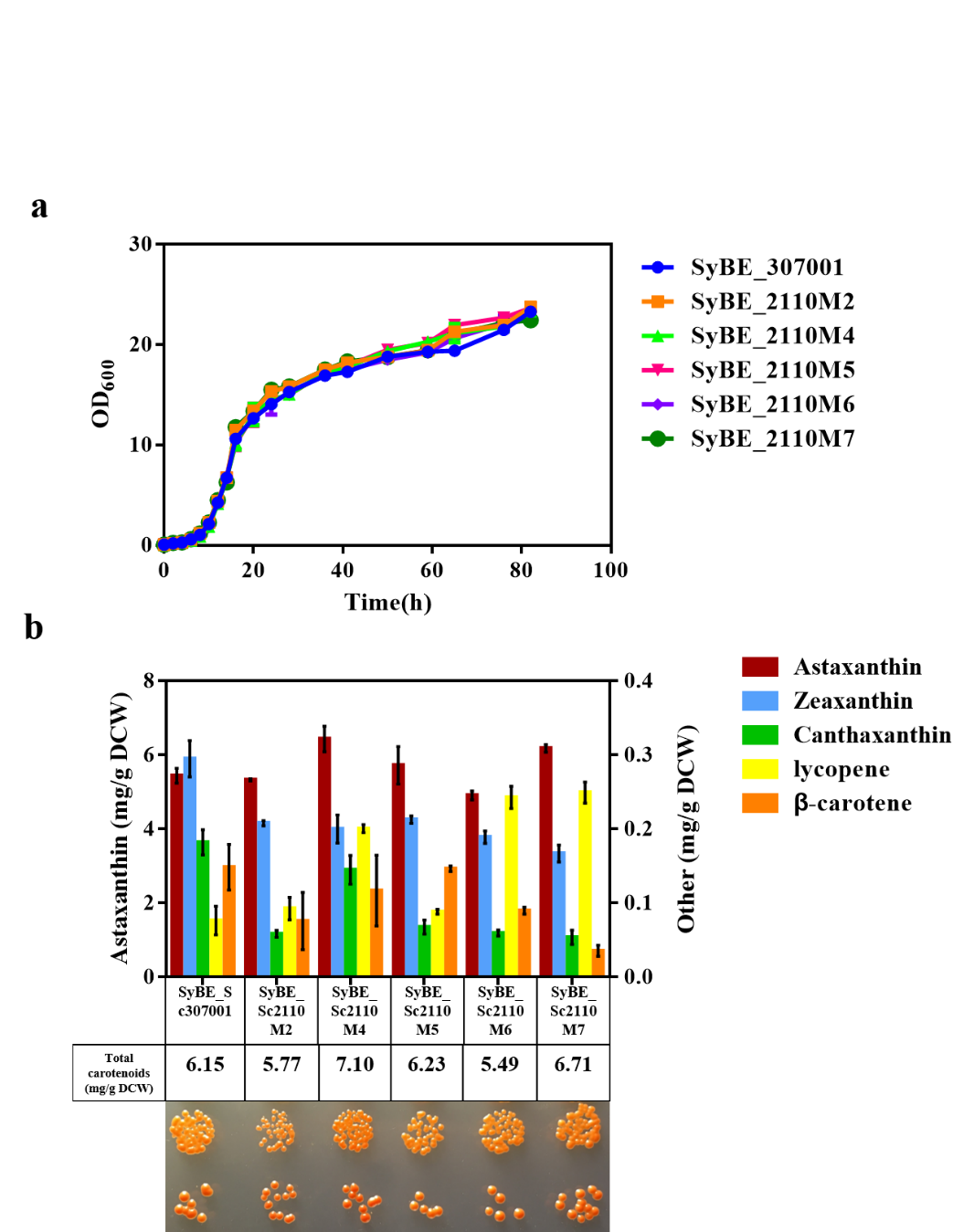


**Figure S4.** Cell growth and astaxanthin yield of ARTP mutants. Growth curve (**a**) and carotenoids profile (**b**) of strain SyBE_Sc307001 and its ARTP mutagenesis strains (SyBE_Sc2110M2, M4-M7) which did not achieve higher total carotenoids accumulation in YPD medium than the parent strain. A photograph was attached bellow the bar chart to illustrate visual color of the related strains. The error bars represent standard deviations calculated from duplicate experiments. The error bars represent standard deviations calculated from duplicate experiments. “Astaxanthin yield” was determined as “the astaxanthin content in single cell” with unit mg/g DCW.


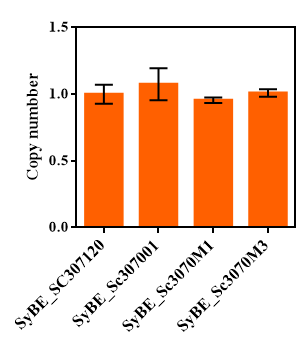


**Figure S5.** The copy numbers of plasmid pRS425K-BDC263_CrtW-Aa_CrtZ in strain SyBE_Sc307001, SyBE_Sc3070M1 and SyBE_Sc3070M3.


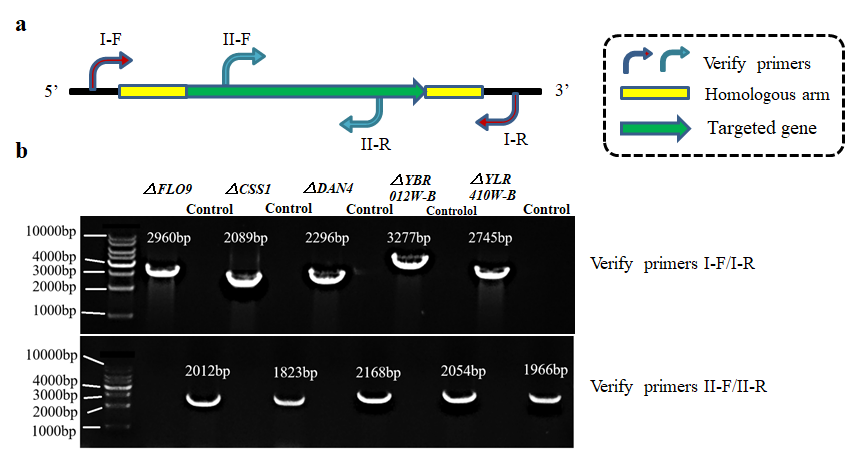


**Figure S6.** PCR verification of the desired gene-deleted strains. (**a**) Sketch map of the design of PCR verify primers. One pair of the verified primers (I-F/I-R) were located upstream of the left homologous arm and downstream of the right homologous arm of the targeted gene; while another pair of the verified primers (II-F/II-R) were located inside of the targeted gene. Primers I-F/I-R are able to amplify a clear band in the DNA poles for gene knocked-out stains; while Primers II-F/II-R are able to amplify a clear band only in the DNA poles for the parent stain. (**b**) Electrophoretic map of PCR products. To be notably, the primers I-F/I-R could amplify the band from control, which should be 5456 bp (for *FLO9*), 4988 bp (for *CSS1*), 4659 bp (for *DAN4*), 6724 bp (for *YBR012W-B*) and 6154 bp (*YLR410W-B*). However, the current extension time was just enough to amplify the band from the gene deleted strains but it was too short (or it was hard) to obtain the band from control.


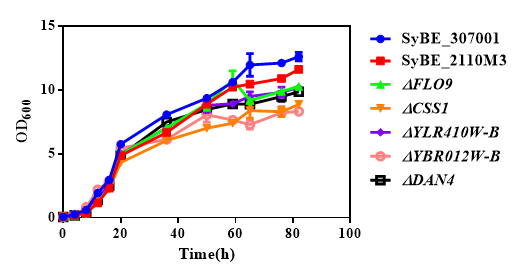


**Figure S7.** Growth curve of strains SyBE_Sc307001, SyBE_Sc2110M3 and gene deleted strains in SC medium. These gene knocked-out strains were generated from strain SyBE_Sc307001 by individual deletion of gene *FLO9, CSS1, YLR410W-B, YBR012W-B and DAN4*, respectively.

**Figure S8.** Astaxanthin yield of stain SyBE_Sc2110M3 in each generation cultivated in SD medium.
